# Comparison of Functional Outcomes of Tibial Plateau Fractures Treated with Nonlocking and Locking Plate Fixations: A Nonrandomized Clinical Trial

**DOI:** 10.1155/2014/324573

**Published:** 2014-03-16

**Authors:** Mohammad Ali Tahririan, Seyyed Hamid Mousavitadi, Mohsen Derakhshan

**Affiliations:** Department of Orthopedics, Kashani Hospital, Isfahan University of Medical Sciences, Isfahan 8174673461, Iran

## Abstract

Fixation of tibial plateau fractures with plate has been widely used. This prospective study was planned to compare locking plate fixation of tibial plateau fracture with nonlocking methods in terms of their functional outcomes. The subjects of the study were selected from consecutive patients suffering from tibial plateau fractures referred to Kashani Hospital in Isfahan, Iran, between 2012 and 2013 and were candidate for surgery. The final included patients were assigned to two groups, those who were treated with locking plate (*n* = 20) and those who were treated with nonlocking plates (*N* = 21). The mean duration of follow-up was 13.4 months (ranging between 10 and 17 months). The mean of knee scores was significantly higher in locking plate group than in nonlocking plate group at the follow-up time (80.20 ± 10.21 versus 72.52 ± 14.75, *P* = 0.039). Also, the mean VAS pain severity score was significantly lower in locking plate group compared with nonlocking plate group (4.45 ± 2.50 versus 6.00 ± 2.59, *P* = 0.046). This study confirmed superiority of the locking plate method over nonlocking plate method with regard to knee score as well as VAS pain score.

## 1. Introduction

Proximal tibial fractures are important fractures involving one of the main weight-bearing joints whose serious injury results in movement and ability dysfunctions [1]. The main goal of the treatment of these fractures is to maintain the normal function of the knee joint, improve the joint instability, prevent lower limb malalignment and deformity, and prevent knee osteoarthritis [2–4]. Applying effective preventive approaches can lead to maintained articular surface, uniform plateau level, and a near normal range of knee joint motion. The main defined criteria in functional assessment of patients with proximal plateau fractures of tibia include knee range of motion, time to achieve union, patient's ability to walk, patient's ability to climb stairs, the pain severity while walking as well as at rest, muscle strength, severity of instability in the knee, and loss of active extension of the knee [5–8].

Unfortunately, there is no gold standard treatment approach for various types of tibial plateau fractures; therefore, different methods have been employed depending on the type of fracture. Tibial plateau fixation with plate especially nonlocking plates has been widely used in recent years [9, 10]. One of the commonly applied types of these plates is the locking compression plate that provides greater stability in these unstable fractures and creates a stronger connection between the articular components [11]. Stabilizing the joint surface by this method, due to its less invasiveness, not only seems to cause a significant decrease in side effects but also reduces the length of hospital stay and hospital costs [12, 13]. The present prospective follow-up study was to compare locking plate fixation of tibial plateau fractures and nonlocking methods in terms of long-term articular functional outcomes.

## 2. Methods

**Figure 1:**
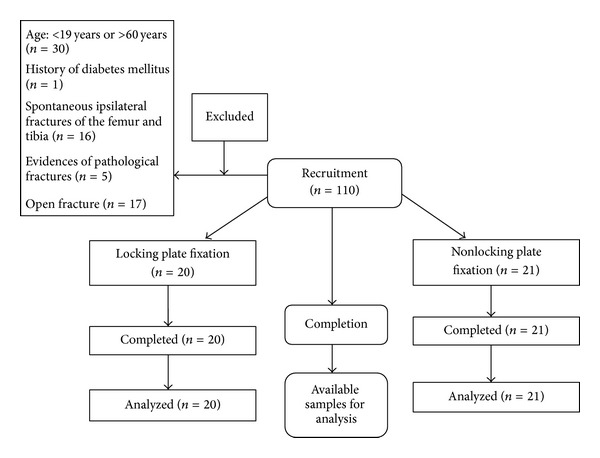
Flowchart of trial.

This prospective nonrandomized clinical trial with a parallel design and allocation ratio of 1 : 1 was conducted to compare success rate and outcomes of locking and nonlocking plate fixation of tibial plateau fractures. The subjects were selected from consecutive patients suffering from tibial plateau fractures and who were referred to Kashani Hospital in Isfahan, Iran, between January 2011 and January 2013 and were candidates for surgery (*n* = 110). All the participants had unilateral closed fractures without open wound or neuromuscular complications. Exclusion criteria in this study were patients younger than 19 years or older than 60 years, history of diabetes mellitus, Ipsilateral fractures of the femur and tibia, pathological fractures, and open fracture (Figure 1). Forty one patients were thus selected for the study. On admission, written consent was acquired from all patients. The selected patients were assigned to two groups; group one was treated with locking plate fixation (*n* = 20) and group two with nonlocking plate fixation (*N* = 21). The patients' assignment to one of the two interventional groups was based on discretion of the physician and also the special condition of the patients for selecting one of the two methods.

All patients underwent control radiography after surgery and reduction in all study subjects were near anatomic. Within the treatment schedule and due to intraoperative bone defect, bone graft was used for 12 patients (3 in locking plate and 9 in nonlocking plate). Also, 2 patients received double plate (nonlocking plate type) in both medial and lateral sides with concomitant bone graft. Postoperatively, all patients received supportive care and were discharged, if possible. The splint was used for 3 to 7 days and knee motion started within 2 weeks. The patients were followed up for 10 to 17 months. The status of the tibial plateau fractures was classified according to the Schatzker and AO classifications systems using available preoperative X-rays or CT scans. The two parameters of step-off and widening of articular surface were assessed before and 6 months after surgery. Postoperative X-rays were assessed according to Freedman and Johnson's description for determining the alignment of the tibial plateau, both on coronal plane (medial proximal tibial angle or MPTA) and sagittal plane (posterior proximal tibial angle or PPTA) [14]. Functional outcomes in ten months were assessed using the Knee Society knee score that considers a clinical score (including pain, stability, range of motion, flexion contracture, extension lag, and malalignment) and a functional score (that assesses walking distance and stair climbing) [15]. This score was graded as excellent (80 to 100), good (70 to 79), fair (60 to 69), and poor (below 60) [16]. The severity of pain was assessed using a visual analogue scale (VAS). For all the patients, the knee range of motion (ROM) was measured using a large goniometer with 25 cm movable arms, marked with one-degree increments.

The sample size was determined at 95% confidence interval (CI), 10% precision, and was based on mean ROM indices between the locking plate and nonlocking plate groups in the previous studies and was found to consist of at least 20 patients in each study groups. In this regard, the study power was also determined at 85.5%. For statistical analysis, categorized variables were compared using chi-square or Fisher exact tests as required. Continuous variables were compared using independent* t*-test and Mann-Whitney* U* test. We used multivariate logistic regression analysis to investigate the potential confounding effects of patients' characteristics and clinical data on the difference in outcomes of surgical protocols. The significance of the results was determined at *P* values of 0.05 or less. All the statistical analyses were performed using SPSS version 19.0 (SPSS Inc., Chicago, IL, USA).

## 3. Results

In this study 20 patients were treated with locking plates and 21 were treated with nonlocking plates. The two intervention groups were similar for mean age (34.50 ± 7.92 years, ranging from 20 to 54 years, versus 34.55 ± 10.34 years, ranging from 21 to 55 years, *P* = 0.986) and male gender distribution (85.0% versus 90.5%, respectively, *P* = 0.663). The average duration of follow-up was a total of 13.4 months (ranging from 10 to 17 months). No difference was observed in the duration of follow-up between the locking plate and nonlocking plate groups (10.61 ± 3.05 months versus 12.95 ± 2.63 months, *P* = 0.158). As shown in Table 1, no differences were noticed in the types and patterns of fractures between nonlocking and locking plate fixation methods based on both Schatzker and AO classifications. In this regard, the most common type of fractures based on the Schatzker system was tibial plateau fracture with spilt depression or type II (52.4% in nonlocking and 45.0% in locking methods) followed by tibial plateau fracture with diaphyseal discontinuity or type VI (19.0% in nonlocking and 25.0% in locking methods) with no significant difference between the two groups (*P* = 0.556). 

No difference was observed in the mean age of the patients with different types of fractures based on the two classification systems, and the most common mechanisms for tibial plateau fracture were motor accident (MA) (41.5%) and motor to car accident (MTCA) (22.0%), respectively.

**Figure 2:**
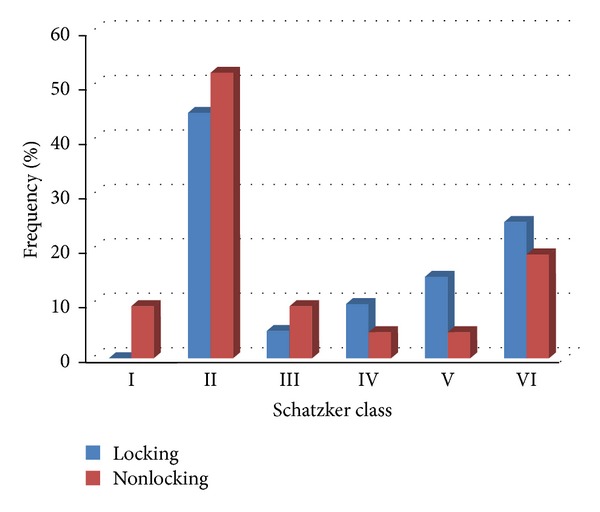
Schatzker classification in two locking and nonlocking groups.

**Figure 3:**
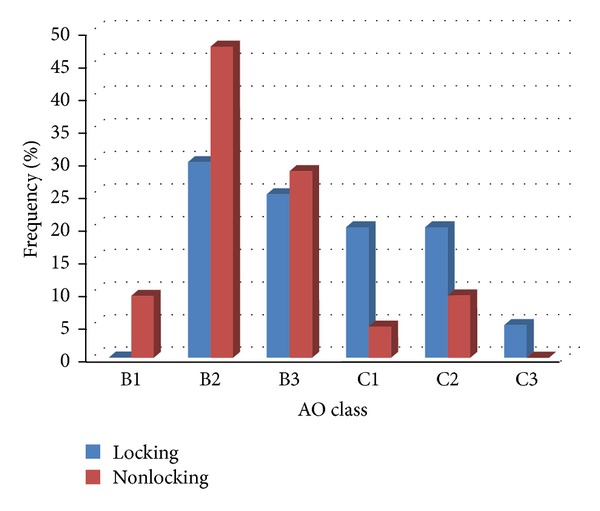
AO classification in two locking and nonlocking groups.

Regarding postoperative complications, the overall complication rate was 17.1% (7 out of 41 patients). One case had a deep wound breakdown that received double-plate and was managed and improved by reoperation, irrigation, wound debridement, and antibiotic therapy. Superficial infection was revealed in 6 patients (1 in the locking plate and 5 in the nonlocking plate groups) who were all successfully treated with antibiotic therapy. There were no cases of compartment syndrome, deep vein thrombosis, or nonunion. No significant differences were found between the two groups in the severity of the tibial plateau fractures according to the Schatzker (*P* = 0.556) or AO (*P* = 0.257) classifications systems (Figures 2 and 3).

**Figure 4:**
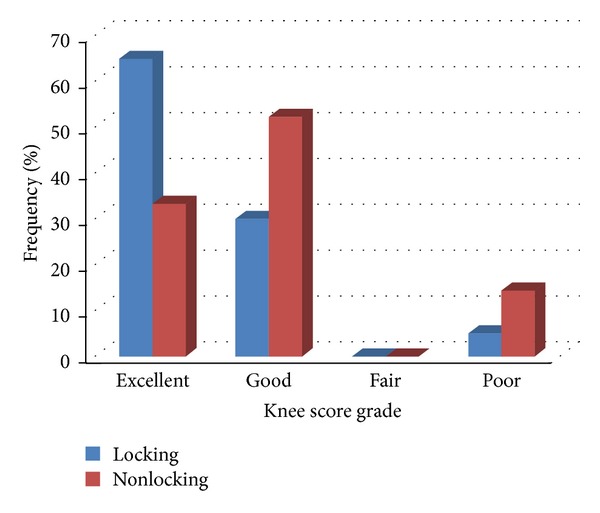
Frequency of knee score grades in two locking and nonlocking groups.

At the final follow-up, a total of 11 cases had step-off of more than 2 mm (4 cases in the locking plate and 7 cases in the nonlocking plate groups). Also, widening of articular surface of more than 2 mm was found in 5 cases (1 case in the locking plate and 4 cases in the nonlocking plate groups). As presented in Table 2, no significant association was noted between postoperative step-off or widening statuses with the type of fracture according to the two fracture classification systems. The MPTA showed a range of 75–100° (mean = 88.73 ± 5.26°). Eleven cases (26.8%) were outside the normal range (82–92°) and were considered malaligned, of whom 11 (9 nonlocking and 2 locking) were with a valgus angulation (i.e., >92°) and one with a varus angulation (i.e., <82°). In the sagittal plane the PPTA demonstrated a range of 1° to 22° (mean = 7.87 ± 5.14°). Seventeen cases (41.5%) were outside the normal range (4–14°) and considered malaligned in the sagittal plane. There were 3 cases with a PPTA > 14° and 14 cases with a PPTA < 4°. Four of these cases were treated with locking plates and 13 cases treated with nonlocking plates. The average range of motion (ROM) was 118.95 ± 17.13° (range of 70–150°). Among the 41 subjects, 9 (22.0%) had a ROM of less than or equal to 100. No difference was observed in the mean of ROM between the locking plate and nonlocking plate groups (122.35 ± 12.93° versus 115.71 ± 20.14°, *P* = 0.219). The mean of knee scores was significantly higher in the locking plate group as compared with the nonlocking plate group at the most recent follow-up (80.20 ± 10.21 versus 72.52 ± 14.75, *P* = 0.039). In this regard, excellent grade of knee score was shown in 65.0% in the locking plate group and in 33.3% in the nonlocking plate group (Figure 4).

Also, the mean of functional score was also higher in the locking plate group than in the nonlocking plate group at the follow-up (77.26 ± 9.95 versus 69.55 ± 10.22, *P* = 0.026). In this regard, excellent grade of functional score was also found in 70.0% in the locking plate group and in 38.1% in the nonlocking plate group.

The mean VAS pain score was significantly lower in the locking plate group compared with the nonlocking plate group (4.45 ± 2.50 versus 6.00 ± 2.59, *P* = 0.046). Postoperative VAS scores positively correlated with both preoperative and postoperative step-off and widening statuses as is shown in Table 3. In this regard, both knee scores and ROM adversely correlated with postoperative step-off and widening status. The inverse relationship between postoperative step-off and widening and the level of knee scores is also shown in Table 4.

## 4. Discussion

The advent and development of locking compression plate method has effectively improved tibial plateau fractures as common complex fractures. Few published studies have compared long-term results of this procedure especially with respect to functional outcomes with nonlocking methods. The present study showed superiority of the locking plate method to the nonlocking plate with regard to knee score and AS pain scores. On the other hand, to improve the knee functional score and minimize postoperative pain, considering locking plate is preferable to nonlocking plate. Although this superiority seems to be preserved in terms of other parameters including ROM index, function score, bone-graft need, and even postoperative complications, because of employing small sample size in our study, no statistically significant differences were witnessed. Other studies with similar sample sizes obtained similar success in the use of locking plates. Stannard and colleagues [17] collected data from a series of 39 tibial plateau fractures, all of which healed without further intervention and with only two superficial wound infections as complications. Cole et al. [18] reported the results of 42 consecutive tibial plateau fractures with 91% union, 9% malalignment, and 4% infection rate. A study by Ricci et al. [19] reported that 37 of 38 fractures healed without complication and with acceptable alignment employing this treatment method. Most recently, a study by Lee et al. [20] also demonstrated results with no loss of reduction, nonunion, and infection developing in only two of 35 fractures. To compare the outcomes of open reduction and locked plating versus fine-wire external fixation of 58 consecutive bicondylar tibial plateau fractures, Krupp et al. [21] found that locked plating was associated with a decreased time to union, decreased incidence of articular malunion, decreased knee stiffness, and decreased overall complications. Moreover, Biggi [22] discovered that internal fixation with locking plates, following the principles of minimally invasive percutaneous osteosynthesis, could provide satisfactory fracture reduction with good results regarding the midterm clinical outcome. Contrarily, Littlechild et al. [23] noticed that no definite advantage was associated with the use of locked plating for high-energy tibial plateau fractures. Usually a locking plate is inserted providing a raft of proximal locking screws to support the articular surface, buttressing the lateral wall of the proximal tibia and extending distally to adequately support the construct. Because the main goal of the treatment is to restore the congruence of the articular surface supporting the tibial plateau cartilage which is usually depressed, to fix the fracture with a stable device, and finally to allow early rehabilitation, the locking plate method can result in achieving main therapeutic goals with appropriate long-term surgical outcome.

A positive point of the study was adjusting age distribution as a potential confounder in the use of locking and nonlocking plates. However, the main limitations of the study included nonrandomized trial, small sample size, and thus partially low study vigor. To confirm advantages of this therapeutic option, further studies should be conducted and its outcome should be compared with other traditional therapeutic methods.

## 5. Conclusion

The present study showed superiority of locking plate to nonlocking plate methods with regard to knee scores and VAS pain scores indicating more improvement in knee functional score and minimizing postoperative pain using the locking plate method.

## Figures and Tables

**Table 1:** Type of fractures and common mechanisms of the fracture.

Type of fracture	Nonlocking	Locking	Mean age (mean ± SD)	Common mechanism frequency (%)
Schatzker system				
Grade I	2 (9.5)*	0 (0.0)*	31.00 ± 11.31	MA (50.0)/F (50.0)
Grade II	11 (52.4)	9 (45.0)	34.37 ± 9.45	MA (35.0)/PTCA (20.0)
Grade III	2 (9.5)	1 (5.0)	28.00 ± 4.00	MTCA (66.7)/MA (33.3)
Grade IV	1 (4.8)	2 (10.0)	33.00 ± 15.13	MTCA (33.3)/MA (33.3)
Grade V	1 (4.8)	3 (15.0)	36.50 ± 7.77	MTCA (50.0)/MA (25.0)
Grade VI	4 (19.0)	5 (25.0)	37.44 ± 8.41	MA (66.7)/MTCA (11.1)
AO system				
B_1_	2 (9.5)	0 (0.0)	31.00 ± 11.31	MA (50.0)/F (50.0)
B_2_	10 (47.0)	6 (30.0)	33.25 ± 6.35	MA (31.3)/MTCA (18.8)
B_3_	6 (28.6)	5 (25.0)	33.60 ± 11.42	MA (45.5)/MTCA (27.3)
C_1_	1 (4.8)	4 (20.0)	34.60 ± 4.63	MA (40.0)/MTCA (40.0)
C_2_	2 (9.5)	4 (20.0)	40.66 ± 9.95	MA (50.0)/MTCA (16.7)
C_3_	0 (0.0)	1 (5.0)	34.00 ± 0.00	MA (100)

MA: motor accident; MTCA: motor to car accident.

Data are presented as number (%).

*Analyses were performed using the chi-square test or Fisher's exact test (all *P* values were more than 0.05).

**Table 2:** Postoperative step-off and widening status by fracture systems.

Type of fracture	Locking methods		Nonlocking methods	
	Step-off >2 mm	Widening >2 mm	Step-off >2 mm	Widening >2 mm
Schatzker system				
Grade I	0 (0.0)*	0 (0.0)*	0 (0.0)	0 (0.0)
Grade II	2 (22.2)	0 (0.0)	5 (45.5)	2 (18.2)
Grade III	1 (100)	1 (100)	1 (50.0)	0 (0.0)
Grade IV	0 (0.0)	0 (0.0)	0 (0.0)	1 (100)
Grade V	1 (33.3)	0 (0.0)	0 (0.0)	1 (100)
Grade VI	0 (0.0)	0 (0.0)	1 (25.0)	0 (0.0)
*P* value	0.191	0.682	0.684	0.748
AO system				
B_1_	0 (0.0)	0 (0.0)	0 (0.0)	0 (0.0)
B_2_	1 (16.7)	0 (0.0)	2 (20.0)	1 (10.0)
B_3_	1 (20.0)	0 (0.0)	5 (83.3)	2 (3.3)
C_1_	1 (25.0)	0 (0.0)	0 (0.0)	1 (100)
C_2_	1 (25.0)	1 (25.0)	0 (0.0)	0 (0.0)
C_3_	0 (0.0)	0 (0.0)	0 (0.0)	0 (0.0)
*P* value	0.981	0.378	0.667	0.164

Data are presented as number (%).

*Analyses were performed using the chi-square test or Fisher's exact test (all *P* values were more than 0.05).

**Table 3:** Correlation of pre- and postoperative step-off and widening with three parameters of VAS pain score, knee score, and ROM.

	VAS	Knee score	ROM
Step-off	0.309 (0.049)	−0.242 (0.127)	−0.290 (0.066)

Step-off (after 10 months)	0.375 (0.016)	−0.390 (0.012)	−0.361 (0.020)

Widening	0.375 (0.016)	−0.279 (0.077)	−0.284 (0.072)

Widening (after)	0.383 (0.014)	−0.341 (0.029)	−0.444 0.004

VAS: visual analogue scale; ROM: range of motion.

Data are presented as *r*-coefficient (*P* value).

*Analyses were performed using Pearson's correlation test.

**Table 4:** The relationship between postoperative step-off and widening and the level of knee score.

Level of knee score	Step-off	Widening
Step-off	<2 mm	>2 mm
Excellent	18 (60.0)	2 (18.2)
Good	10 (33.3)	7 (63.6)
Poor	2 (6.7)	2 (18.2)
*P* value	**0.041**	

Widening	<2 mm	>2 mm
Excellent	20 (55.6)	0 (0.0)
Good	13 (36.1)	4 (80.0)
Poor	3 (8.3)	1 (20.0)
*P* value	**0.039**	
